# The Comorbidity of Reduplicative Paramnesia, Intermetamorphosis, Reverse-Intermetamorphosis, Misidentification of Reflection, and Capgras Syndrome in an Adolescent Patient

**DOI:** 10.1155/2014/360480

**Published:** 2014-09-23

**Authors:** Ozden Arısoy, A. Evren Tufan, Rabia Bilici, Sarper Taskiran, Zehra Topal, Nuran Demir, M. Akif Cansız

**Affiliations:** ^1^Department of Psychiatry, Abant Izzet Baysal University Medical Faculty, 14280 Bolu, Turkey; ^2^Department of Child and Adolescent Psychiatry, Abant Izzet Baysal University Medical Faculty, 14280 Bolu, Turkey; ^3^Erenkoy Hospital for Mental Disorders, 34738 Istanbul, Turkey; ^4^Department of Psychiatry, Koc University Medical Faculty, 34738 Istanbul, Turkey

## Abstract

Delusional misidentification syndromes may be superimposed on neurological or psychiatric disorders and include delusional beliefs that the people, objects, or places around the patient change or are made to change with one another. In this paper, an adolescent patient displaying Capgras syndrome, metamorphosis, reverse-intermetamorphosis, misidentification of reflection, and reduplicative paramnesia was presented. The findings that our patient struggled with visuospatial tests applied in the acute phase as well as the observation that she refused to meet her family face-to-face while accepting to speak on the phone may support the role of right hemisphere and visuospatial functions in the development of those syndromes. Further studies or case series evaluated more extensively are needed to reveal the relationship between right hemisphere functions and delusional misidentification syndromes.

## 1. Introduction

“Delusional misidentification syndromes” may be superimposed on neurological or psychiatric disorders and include delusional beliefs that the people, objects, or places around the patient change or are made to change with one another. They can be divided into Capgras and Fregoli delusions as well as intermetamorphosis and twinning (döppelgänger). Rarer phenomena such as misidentification of self in the mirror, reduplicative paramnesia, and clonal multiplication of the self are also listed among those syndromes. Capgras syndrome involves a belief that the patients' acquaintances (especially close relatives) are replaced with exact look-alikes. On the other hand, a patient with reduplicative paramnesia believes that a person, a place, or an event around himself/herself to be copied exactly. Intermetamorphosis involves a belief that people around the patient exchange places entirely, not limiting themselves to disguises or superficial appearances. Reverse-intermetamorphosis involves a belief that another person replaced the patient both physically and mentally while misidentification of reflection involves a belief that the reflection in the mirror is not of the patient but of a different person resembling the patient [1]. Here, we present an adolescent patient with Capgras syndrome, intermetamorphosis, reverse-intermetamorphosis, misidentification of reflection, and reduplicative paramnesia. The patient was deemed to be worthy of presentation both due to the rarity of those syndromes in adolescence and for being evaluated with neuropsychological tests.

## 2. Case

The patient was a 17-year-old female high school senior who was brought to our department with complaints of “rambling and incoherent speech, running away from home, and believing that her family members were actually imposters trying to persecute her.” Upon questioning it was revealed that her complaints started three years ago and continued without remission and that she was hospitalized twice for a total of 51 days with a diagnosis of schizoaffective disorder. She was treated with risperidone 6 mg/day, quetiapine 300 mg/day, and valproate 750 mg/day for varying durations but her compliance was poor and there was no benefit from drugs. The complaints increased a month before her last application. Family history revealed that her mother was diagnosed with schizoaffective disorder and died after a suicide attempt fifteen years ago and that her maternal uncle was diagnosed and treated with psychotic disorder, not otherwise specified. The patient was promptly hospitalized for differential diagnosis and treatment. Baseline mental status examination showed reduced grooming and excessive suspiciousness. Mood and affect were irritable. Speech was pressured. Thought processes were incoherent and the content was bizarre. She reported that other people replaced her family and that her “true” family was in another city (Capgras syndrome), that her father had 250 wives but she knew only one of them, that the hospital in which she was being evaluated was in another city (reduplicative paramnesia), that her father was trying to harm her (persecutory delusion), that ward personnel, nurses, and other patients regularly change places between themselves and with objects (intermetamorphosis), that one of the nurses changed places with her via “magic” and their physical features changed as well (reverse-intermetamorphosis), that her reflection in the mirror was actually that of another girl, resembling herself (misidentification of reflection), and that her father was actually God (grandiosity). Psychomotor activity was increased while insight was lacking.

Psychometric evaluations revealed scores of 107 (positive: 36, negative: 23, and general psychopathology: 48) and 24, respectively, from Positive and Negative Syndrome Scale (PANSS) and Mini-Mental Status Examination (MMSE). She was noted to have problems in copying figures and doing calculations and in immediate recall. Visuomotor evaluation with Bender-Gestalt test revealed an error score of 9 points. Physical and neurological examinations and laboratory evaluations including toxicology screen were normal. Crainal MRI and EEG were within normal limits. As a result, the patient was diagnosed with schizoaffective disorder-manic type according to DSM-IV-TR criteria. Due to low compliance the patient was started on parenteral risperidone 37.5 mg/every 15 days. Risperidone 6 mg/day and valproate 750 mg/day orally were continued until the 3rd week. During hospitalization the patient refused meetings with her family while she had no problems in receiving telephone calls from them. Evaluation at the 6th week of hospitalization revealed a PANSS score of 65 (positive: 21, negative: 15, and general psychopathology: 29) and a MMSE score of 27. She was noted to have problems in figure copying and doing calculations while her immediate recall was normal. A repeat of Bender-Gestalt test revealed an error score of 5. The patient was discharged on the 60th day with a treatment regimen of parenteral risperidone 37.5 mg/every 15 days.

## 3. Discussion

Here, an adolescent patient with Capgras syndrome, intermetamorphosis, reverse-intermetamorphosis, misidentification of reflection, and reduplicative paramnesia in combination was presented. Previously an adolescent patient displaying Capgras, Fregoli, and Cotard syndromes together was presented [2] while other reports mostly focused on isolated Capgras syndrome in adolescent patients [3–7]. As far as we are aware, this is the first report of an adolescent patient displaying a variety of delusional misidentification syndromes together.

Capgras syndrome is reported to be the most common of the delusional misidentification syndromes while others are rarer. Those syndromes are reported mostly after right hemispheric lesions. It was also reported that an organic etiology may be found among 25–50% of patients with Capgras syndrome [1–7]. However, no organic etiology could be found in our patient. Previous reports of adolescent patients were also negative for organic etiology. It was previously posited that organic etiologies may be more important for adult patients with those syndromes [2–7].

Right hemisphere is reported to play important roles in visuospatial functions, spatial organization, facial recognition, and emotional and gestalt processing and as such it may play a role in misidentification syndromes [1, 8–11]. Our patient struggled with Bender-Gestalt test and items of MMSE tapping visuospatial functions and she was able to converse with family members on the phone while refusing face-to-face meeting. It was also observed in our patient that visuospatial functions improved albeit partially with antipsychotic treatment and that this correlated with improvement in psychotic symptoms including those of delusional misidentification. Those findings may support the role of right hemisphere in delusional misidentification syndromes.

The visual system is composed of two pathways, one connected with the visual cortex and the other connected with the limbic system. The latter pathway is thought to attribute affective valence to visual stimuli. The delusional misidentification syndromes may arise with problems in connections between visual system and the limbic system. This may result in discordant affective valences being attributed to familiar faces and as such those syndromes may actually be a coping mechanism of the patient to solve this discrepancy [1, 8–11]. It is also known that right hemisphere is more closely related with the limbic system [8]. Bender-Gestalt test involves geometric shapes without affective valence and as such should result in normal scores in our patient according to this hypothesis. However, our patient struggled with this test. This may argue against the hypothesis posited while her refusing face-to-face meetings with family members may support it.

Future reports of delusional misidentification syndromes among adolescent patients preferably involving multiple neuropsychological tests as well as testing with photographs of family members may help clarify those hypotheses.
